# Secondary-Ion Mass Spectrometry of Genetically Encoded Targets**

**DOI:** 10.1002/anie.201411692

**Published:** 2015-03-17

**Authors:** Ingrid C Vreja, Selda Kabatas, Sinem K Saka, Katharina Kröhnert, Carmen Höschen, Felipe Opazo, Ulf Diederichsen, Silvio O Rizzoli

**Affiliations:** Department of Neuro- and Sensory Physiology, University Medical Center GöttingenHumboldtallee 23, 37073 Göttingen (Germany); Institute for Organic and Biomolecular Chemistry, University of GöttingenTammannstrasse 2, 37077 Göttingen (Germany); Department of Ecology and Ecosystem Management, Center of Life and Food Sciences Weihenstephan, Technische Universität MünchenFreising-Weihenstephan (Germany); Center for Nanoscale Microscopy and Molecular Physiology of the Brain (CNMPB)Göttingen (Germany); International Max Planck Research School Molecular BiologyGöttingen (Germany)

**Keywords:** click chemistry, isotopic labeling, protein engineering, secondary-ion mass spectrometry (SIMS), unnatural amino acid

## Abstract

Secondary ion mass spectrometry (SIMS) is generally used in imaging the isotopic composition of various materials. It is becoming increasingly popular in biology, especially for investigations of cellular metabolism. However, individual proteins are difficult to identify in SIMS, which limits the ability of this technology to study individual compartments or protein complexes. We present a method for *s*pecific protein isotopic and fluorescence labeling (SPILL), based on a novel click reaction with isotopic probes. Using this method, we added ^19^F-enriched labels to different proteins, and visualized them by NanoSIMS and fluorescence microscopy. The ^19^F signal allowed the precise visualization of the protein of interest, with minimal background, and enabled correlative studies of protein distribution and cellular metabolism or composition. SPILL can be applied to biological systems suitable for click chemistry, which include most cell-culture systems, as well as small model organisms.

A number of technologies have been recently developed to investigate the chemical composition of biological samples, including matrix-assisted laser desorption/ionization (MALDI),[1,2] laser-ablation inductively coupled plasma mass spectrometry (LA-ICP-MS),[3] or secondary-ion mass spectrometry (SIMS).[4,5] SIMS has the highest spatial resolution, approximately 30–50 nm in the lateral direction (*x*–*y* plane), and down to 5–10 nm in the axial direction (*z* direction), when operating in the NanoSIMS configuration.[5] NanoSIMS is based on the use of a primary ion beam, such as Cs^+^ ions, which is scanned across the surface of the sample, where it causes the release of secondary ions. The isotopic nature of the released ions is analyzed by mass spectrometry, thereby providing an image of the sample composition.

NanoSIMS experiments are often descriptive, measuring the overall distribution of native isotopes in the samples.[6,7] Other applications are based on introducing a non-native, isotopically labeled molecule into the biological sample, and measuring its distribution.[8,9] A related procedure measures cellular turnover, by pulsing cells with an isotopically labeled metabolite, such as an amino acid or a nucleotide, and imaging its incorporation.[10–12]

However, all of these applications suffer from one major limitation: it is difficult to identify specific proteins or specific organelles, except for a few that can be identified by their morphology.[4,10] One proposed solution has been the correlation of NanoSIMS with fluorescence imaging.[4] This approach is difficult, since it requires the use of two instruments, and a very precise overlap of the two types of images. Another solution has been to immunostain the samples using antibodies coupled to isotopically pure metals, such as lanthanides.[13] These antibodies can be imaged in NanoSIMS, without the need for correlative microscopy. This technique, however, undermines to some extent the high resolution of NanoSIMS, since antibodies, and especially the ones coupled to large metal tags, incorporate relatively poorly into specimens, and find only a small fraction of the epitopes, thus resulting in a less-precise image.[14,15]

This type of problem has been solved in fluorescence imaging by the introduction of green fluorescent proteins (GFPs), which can be coupled to native proteins by genetic encoding. We sought to perform a comparable strategy for NanoSIMS imaging by introducing a specific isotope into the protein of interest. Three major conditions are required. First, the genetic encoding procedure should be sufficiently flexible to enable the introduction of the isotope in any protein, in as many types of biological samples as possible. Second, the isotope should be linked to the protein of interest through a highly specific reaction. Third, the isotope should not be present at high levels in untreated preparations, to enable a high signal-to-noise ratio.

The first and the second condition are best fulfilled by what is known as genetic-code expansion, and subsequent click-chemistry labeling (see Figure 1). This technique incorporates unnatural amino acids (UAAs) into the proteins of interest.[16,17] The UAAs are then revealed by coupling to different probes, such as fluorophores, for example through the copper-catalyzed azide–alkyne cycloaddition (click chemistry),[18] or its copper-free variants. To incorporate the UAA into a specific protein, a modified version of the protein containing an Amber stop codon (TAG) is expressed together with a pair of suitable tRNA and aminoacyl–tRNA synthetase (tRNA/RS). The tRNA/RS pair introduces the UAA selectively into the protein of interest, at the site determined by the Amber codon.[19,20] A major advantage of click chemistry over other genetic encoding procedures is that the tag, the alkyne or azide moiety present on the UAA, is extremely small, of only a few atoms in size. It is therefore less bulky than tags such as GFP, and offers several other technical advantages.[21] The coupling of specific fluorophores or isotopes to the UAAs takes place only after cellular fixation, when functional perturbations are no longer a concern. In principle, the experiment could even be performed without the subsequent click-chemistry reaction, by incorporating amino acids containing atoms or isotopes that are not naturally abundant in cells, such as selenocysteine,[22] albeit this only allows the incorporation of one Se isotope per protein, which would result in a fairly low signal-to-noise ratio.

**Figure 1:**
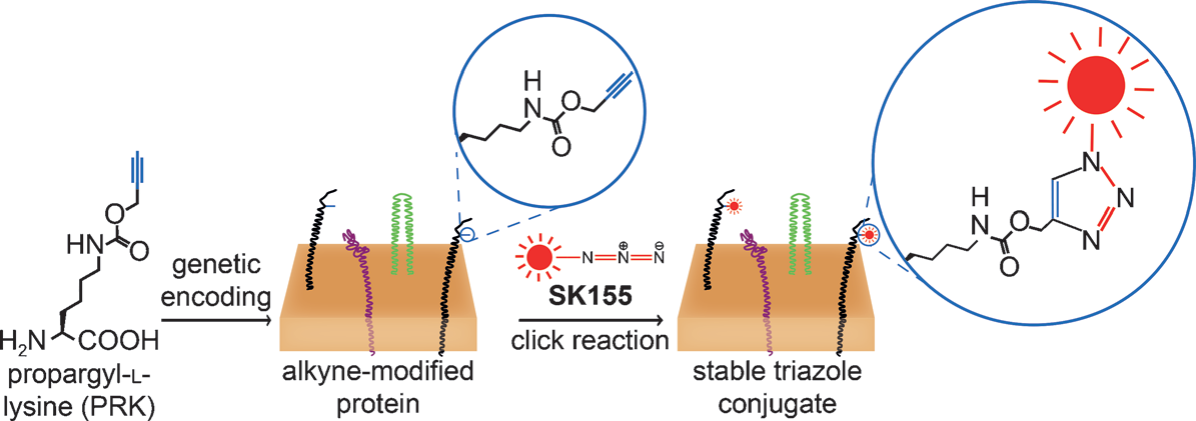
Propargyl-l-lysine (PRK) incorporation into proteins of interest and click reaction with SK155, a dual probe that can be used in both fluorescence and SIMS imaging.

We tested the specificity of the click-based labeling in mammalian cells, by expressing tagged versions of several proteins involved in membrane fusion (SNAREs): the transmembrane syntaxin 1 and syntaxin 13, and the membrane-attached SNAP-25 (Figure S1 in the Supporting Information),[23] which we chose based on our experience with their biology and tagging.[24,25] The UAA propargyl-l-lysine (PRK), which contains an alkyne group, was introduced relying on the wild-type tRNA/RS pair from *Methanosarcina mazei*.[19,20] All three proteins were detected with high specificity by this procedure (Figure S2).

To identify the proteins in NanoSIMS, we sought to couple an isotopically enriched probe to the alkyne group of the PRK (Figure 1). We found ^19^F to be easily identified in NanoSIMS, and to be present at low levels in normal cells, and should thus provide a high signal-to-noise ratio. We therefore produced a clickable dipentafluorobenzyl peptide containing 13 ^19^F atoms, which we termed **SK155** (Figure 2). To enable convenient testing of this probe by fluorescence microscopy, we also introduced the fluorophore Abberior Star635, which contributes three of the ^19^F atoms.[26] A nona-peptide sequence was designed to provide proper solubility under physiological conditions, by incorporating four negatively charged amino acids (glutamate and aspartate). Lysine amino acids with orthogonal protection were used for later side-chain attachment of the Star635-NHS fluorophore (Abberior GmbH, Germany), and an N-terminal lysine served for the linkage of two 2,3,4,5,6-pentafluorobenzoic acid units. Finally, azidolysine (Fmoc-Lys(N_3_)-OH) was included, providing the azide for later attachment of the label **SK155** to proteins by click chemistry. Synthesis of **SK155** (Figure S3) was provided by microwave-mediated solid-phase peptide synthesis (SPPS) of the nona-peptide on a Sieber amide resin, applying the Fmoc synthesis method with O-(benzotriazol-1-yl)-*N*,*N*,*N*′,*N*′-tetra-methyluronium hexafluorophosphate (HBTU), 1-hydroxy-benzotriazole (HOBt) activation. The 2,3,4,5,6-pentafluorobenzoic acid units were coupled on solid support.[27] Cleavage from the resin provided the nona-peptide (C_6_F_5_CO)_2_-Lys-Asp-Glu-Lys(N_3_)-Gly-Asp-Glu-Lys-Gly-NH_2_ that was coupled to the Star635-NHS in solution. After purification by HPLC, the constitutional integrity of the label **SK155** was indicated by high-resolution mass spectrometry.

**Figure 2:**
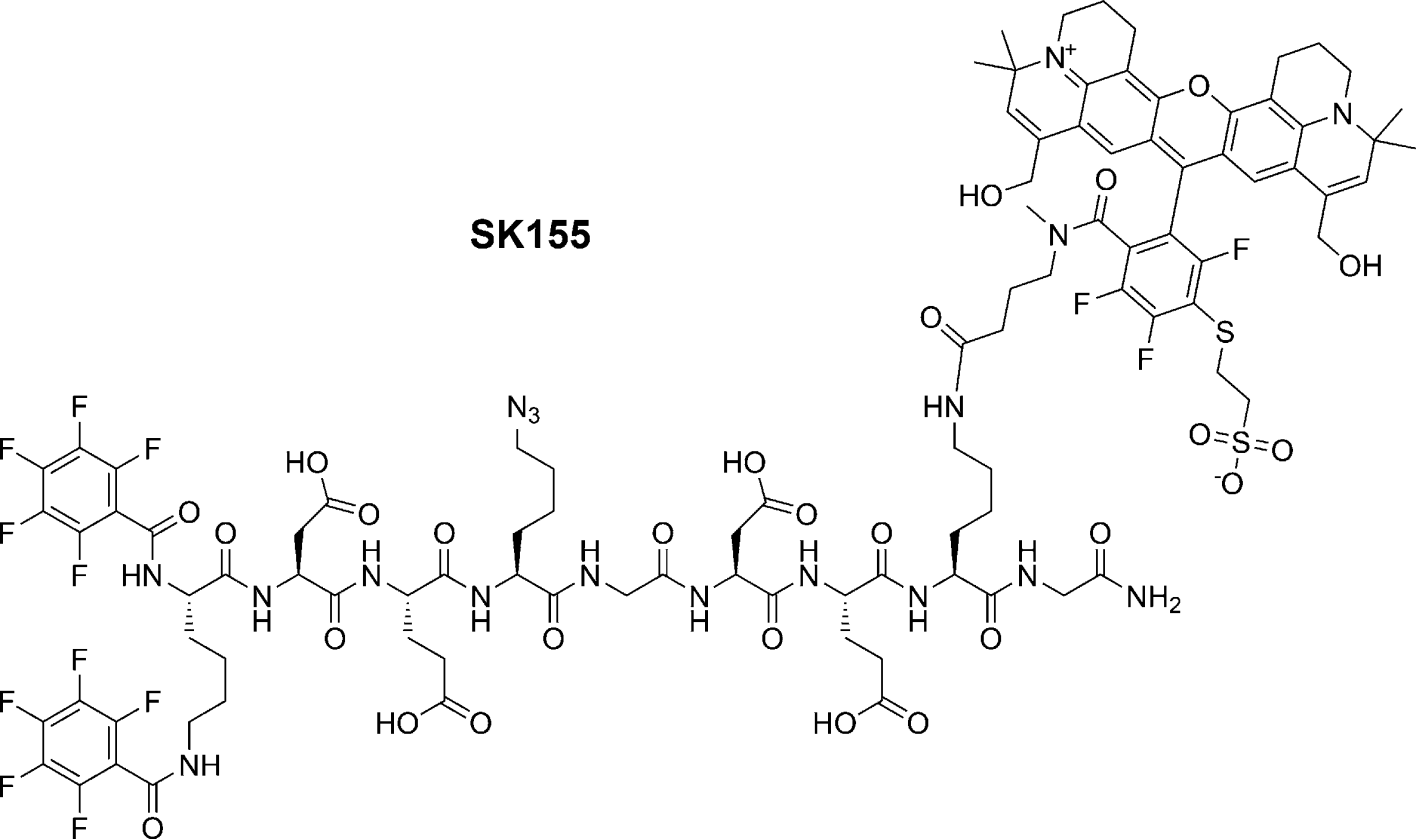
SK155 contains ^19^F atoms as isotopic labels for SIMS, and an Abberior Star635 fluorescent moiety.

The ^19^F distribution overlapped well with the fluorescence signal (Figure 3 A and Figure S4), as expected, and also enabled the analysis of various other parameters, both in the fluorescence and in the isotopic domains (Figure 3 A). The labeling procedure was efficient (Figure 3 B), and the ^19^F signal was found, as expected, within the areas containing the fluorescence signal associated with the protein of interest (Figure 3 C).

**Figure 3:**
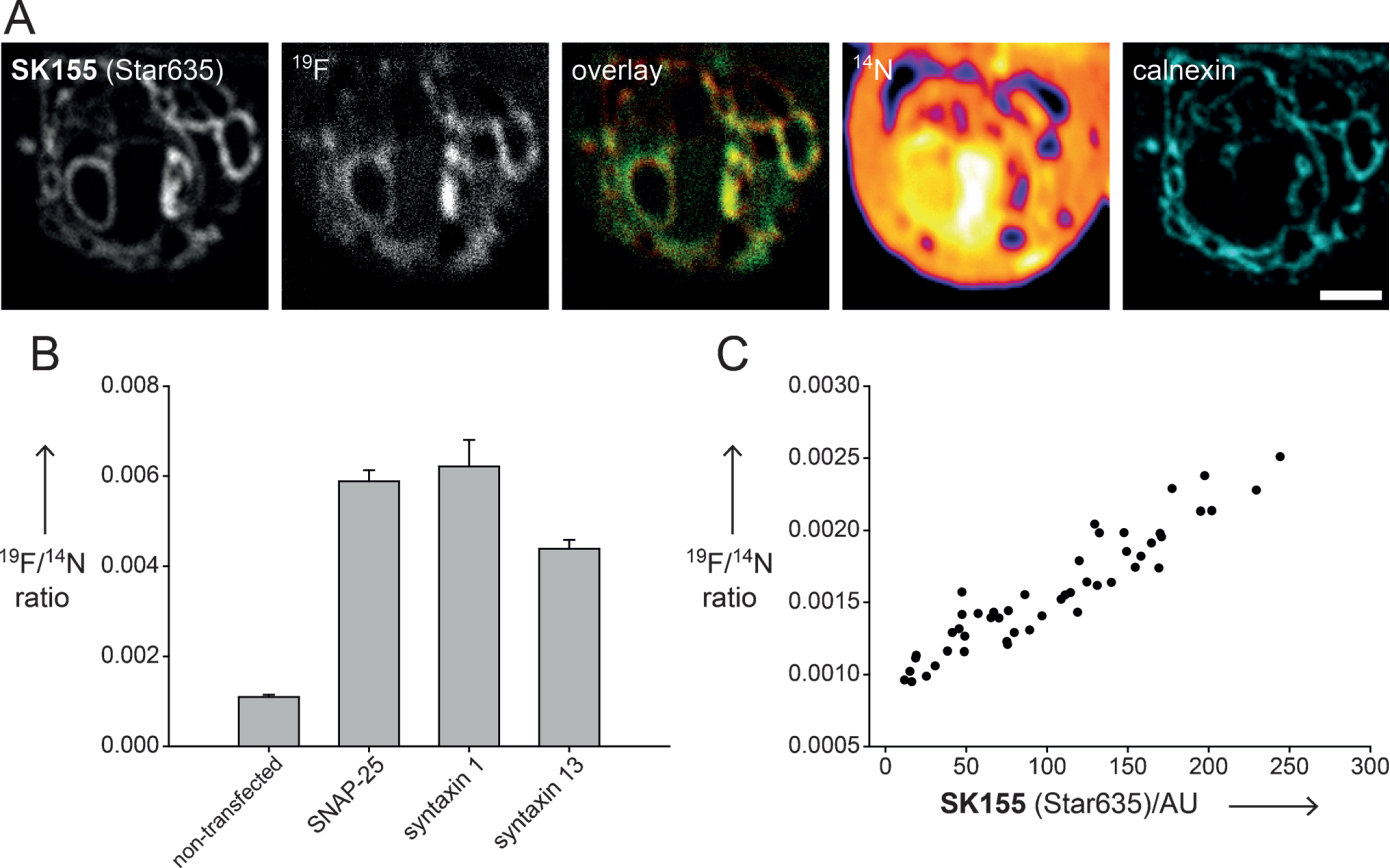
Genetic incorporation of unnatural amino acids (UAAs) as a tool to label proteins for NanoSIMS. A) Fluorescence and NanoSIMS images of a representative cell that had incorporated PRK in syntaxin 1, and was subsequently labeled with SK155. A confocal image of the Star635 fluorescence (left), a ^19^F NanoSIMS image, and an overlay (Star635 signal in red; ^19^F in green). Additional fluorescence and NanoSIMS images are shown: a ^14^N image, and a confocal image of immunostained calnexin, an endoplasmic reticulum marker. Scale bar: 2 μm. B) The ratio between ^19^F and ^14^N in non-transfected cells was measured and compared with cells expressing different proteins. It was significantly higher for all expressed proteins: SNAP-25 (*p*<0.01), syntaxin 1 (*p*<0.001), and syntaxin 13 (*p*<0.001; t-tests). The number of analyzed cellular regions is: 32 for non-transfected, 371 for SNAP-25, 281 for syntaxin 1, and 448 for syntaxin 13 expressing cells. C) Linear relationship between ^19^F/^14^N ratio in a cell and SK155 (Star635) intensity in the corresponding cell regions.

These experiments indicate that *s*pecific protein isotopic and fluorescence labeling (SPILL) is feasible. SPILL offers two major advantages for NanoSIMS imaging. First, it alleviates the need for correlation with fluorescence microscopy to investigate specific proteins. Second, it enables the investigator to use the high axial resolution of NanoSIMS. When employing a correlation with fluorescence imaging, the axial resolution of the correlated images is determined by the optical technique, and is typically 50-fold lower than the axial resolution of NanoSIMS.[4] This problem is avoided using SPILL.

In a proof-of-principle experiment, we combined SPILL imaging with an analysis of cellular turnover, relying on the incorporation of isotopically labeled metabolites. We treated the cells with ^15^N-labeled leucine, which is incorporated into all newly translated proteins, and thus indicates the turnover of the protein-based cellular structures (see Figure S5 for a timeline of the experiments). The ratio between the ^15^N and ^14^N isotopes provides an accurate measurement of turnover: the higher this ratio, the higher the incorporation of new proteins within the particular structure (Figure 4 A). Relying on the high axial resolution of NanoSIMS, we probed the ^15^N, ^14^N, and ^19^F composition of the sample at different depths (see syntaxin 1 images in Figure 4 A), which enabled us to obtain much more information than a simple correlation with the fluorescence image (Figure 4 A, inset). We measured the protein metabolism of the cells at sites marked by the proteins of interest (identified by the high ^19^F signal). Interestingly, our measurements indicated that regions of high protein expression correlated negatively with the ^15^N/^14^N ratio, for both SNAP-25 and syntaxin 13 (Figure 4 B,D), but not for syntaxin 1 (Figure 4 C). Owing to problems in correlating images with different axial resolutions, no significant trends could be observed when comparing the fluorescence signal of the proteins to an average ^15^N/^14^N ratio image obtained by overlapping the images taken at different depths (data not shown). This also constitutes an advantage of NanoSIMS imaging over techniques such as stimulated Raman scattering,[28] which measures turnover with a far lower axial resolution. Albeit, unlike NanoSIMS, these methods are non-invasive, and allow the imaging of living cells.

**Figure 4:**
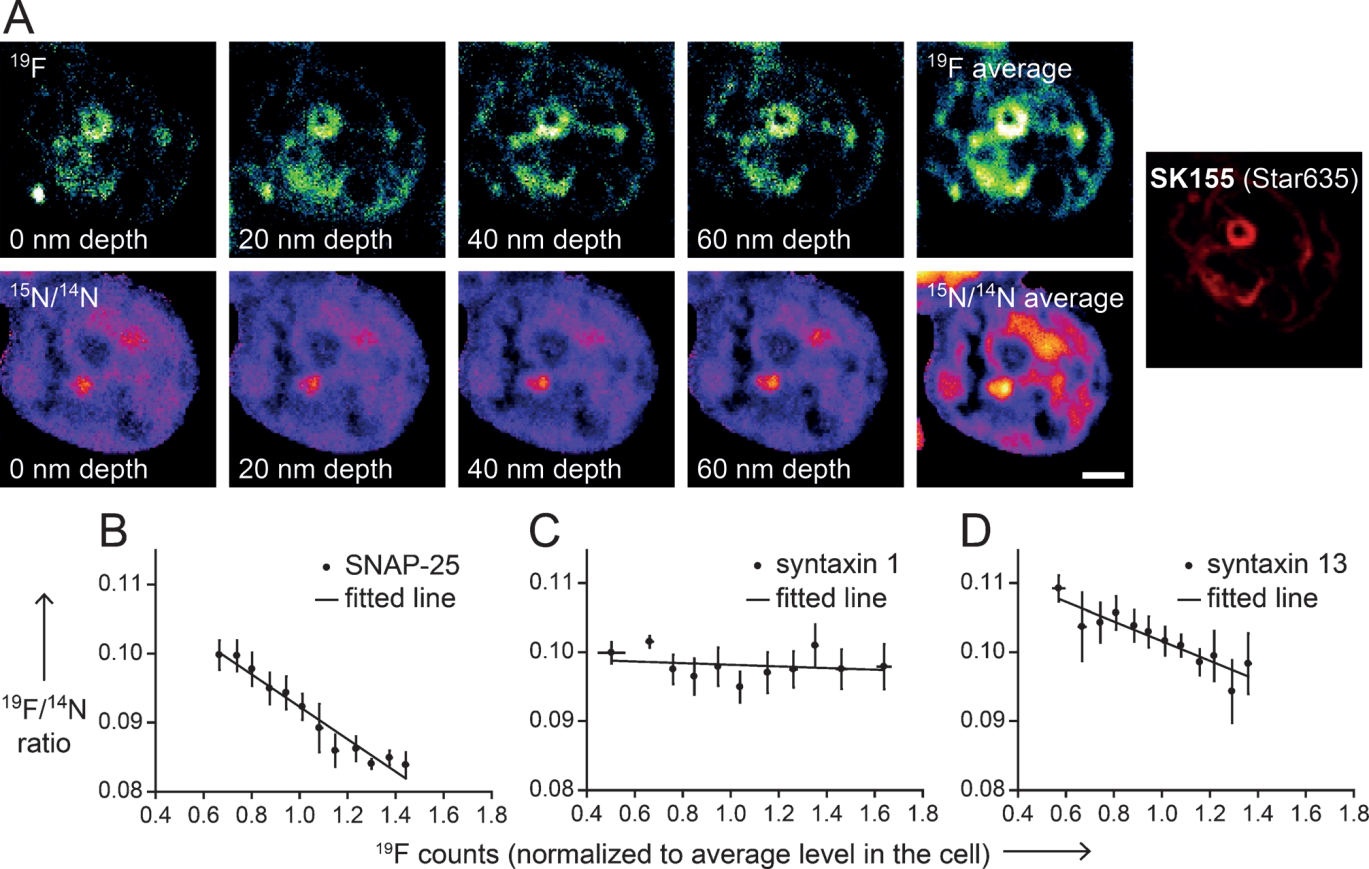
Genetic labeling of proteins and SIMS imaging offer detailed information on how protein metabolism correlates with the presence of a specific protein. A) A series of NanoSIMS images taken at different depth planes in a SK155-labeled cell expressing syntaxin 1. ^19^F is shown on the upper row; the ^15^N/^14^N ratio on the lower row. Inset: for comparison, the fluorescent signal of SK155 in the same cell. Scale bar: 2 μm. B) An analysis of ^15^N/^14^N ratios as a function of ^19^F levels. The number of analyzed cellular regions is 371 for SNAP-25, 281 for syntaxin 1, and 448 for syntaxin 13 samples. A downward trend is observed for all three types of staining, which is statistically significant for SNAP-25 and syntaxin 13 (*p*<0.01, t-tests), but not for syntaxin 1.

The results suggest that the plasma membrane and the early endosomes, where SNAP-25 and syntaxin 13 are located, have a slower metabolism than other parts of the cell. The turnover of the endoplasmic reticulum, where syntaxin 1 is trapped in BHK cells,[29] appears to be similar to the average cell turnover. While the biological significance of these findings awaits testing in other cellular systems, we conclude that the SPILL procedure is an efficient tool for the SIMS-based investigation of specific proteins. The requirements for SPILL are simple: the biological system should allow UAA encoding and click chemistry. This has been already achieved in bacteria, yeast, mammalian cells, *Caenorhabditis elegans*,[30] and *Drosophila melanogaster*,[31] which implies that a large field of experimentation is open to this technology.
