# Supplementary material for: Secondary-Ion Mass Spectrometry of Genetically Encoded Targets
Source: Angew Chem Int Ed Engl. 2015 Mar 17;54(19):5784–8. doi: 10.1002/anie.201411692 (PMC4471591; doi:10.1002/anie.201411692)
Supplement: Supplementary file 1 [file anie0054-5784-sd1.pdf]

Angewandte Chemie
Eine Zeitschrift der Gesellschaft Deutscher Chemiker

Supporting Information

German Edition: DOI:

Secondary-Ion Mass Spectrometry of Genetically Encoded Targets**

Ingrid C. Vreja, Selda Kabatas, Sinem K. Saka, Katharina Kröhnert, Carmen Höschen, Felipe Opazo, Ulf Diederichsen, and Silvio O. Rizzoli**

anie_201411692_sm_miscellaneous_information.pdf

SUPPORTING INFORMATION

Materials for chemical synthesis

Starting materials and reagents were obtained from commercially available sources and were used as supplied. Anhydrous DMF of extra dry or puriss absolute grade (over molecular sieves) was obtained from Sigma-Aldrich (Schnelldorf, Germany). Deuterated DMSO was purchased from Deutero (Kastellaun, Germany). Abberior Star635-NHS ester was obtained from Abberior (Göttingen, Germany). Flash column chromatography on silica was performed using Macherey-Nagel Silica Gel 60 (particle size: 0.063–0.2 mm). Fmoc-protected amino acids, coupling reagents and Sieber amide resin were purchased from GL Biochem (Shanghai) Ltd (Shangai, China). The azide transfer reagent 1*H*-Imidazole-1-sulfonyl azide hydrochloride (**1**) and the azido amino acid (*S*)-2-((((9*H*-fluoren-9-yl)methoxy)carbonyl)amino)-6-azidohexanoic acid (Fmoc-Lys(N_3)-OH, **2**) were prepared as described in literature.[1–3]

Materials for biochemical and cellular experiments

Unless otherwise stated, the chemicals used in this study were purchased either from VWR (Hannover, Germany) or from Sigma-Aldrich Chemie GmbH (Schnelldorf, Germany). Propargyl-L-lysine (PRK) was purchased from Sirius Fine Chemicals SiChem GmbH (Bremen, Germany). Abberior Star635P-azide[4] was provided by Dr. Vladimir Belov, Department of NanoBiophotonics, Max Planck Institute for Biophysical Chemistry, Göttingen, Germany. Abberior Star635-NHS ester[4] was purchased from Abberior GmbH (Göttingen, Germany).

Instruments

NMR spectra were recorded with Varian instruments (Mercury 300, Unity 300 and INOVA-500). ESI mass spectra were recorded with a Finnigan LCQ 7000 spectrometer and a Bruker micrOTOF spectrometer. High-resolution mass spectra (HRMS-ESI) were obtained with a Bruker Apex-Q IV FT-ICR-MS instrument (Breen, Germany). HPLC was performed on a Pharmacia Äkta Basic system (GE Healthcare, London, UK) with a pump-type P-903, variable wavelength detector UV-900 using a Macherey-Nagel Nucleodur® RP C-18 analytical HPLC column (250 x 4.6 mm, 5 µm) and a semipreparative HPLC column (250 x 10 mm, 5 µm). Water for HPLC was purified using a Simplicity water purification system from Millipore. Freeze-drying of compounds from aqueous solutions containing

minimal amounts of MeOH or CH_3CN was performed using a Christ-Alpha-2-4 lyophilizer attached to a high vacuum pump and a Christ RCV-2-18 ultracentrifuge. UV-Spectra for determining the concentration of fluorophore-labeled peptides were recorded with a Thermo Scientific Nanodrop 2000c.

Cells. Baby hamster kidney (BHK) fibroblasts were used for the expression of SNAP-25, syntaxin 1 and syntaxin 13. BHK cells were cultured in Dulbecco's Modified Eagle Medium (DMEM, Life Technologies, Darmstadt, Germany) supplemented with 10% tryptose phosphate, 5% fetal calf serum (FCS), 2 mM L-glutamine, 60 U/mL penicillin, and 60 U/mL streptomycin.

Constructs. The vector pCMV t-RNA-PylRS WT was a gift from Dr. Edward Lemke (EMBL, Heidelberg) and was used as previously described.[5]

For the fluorescent protein constructs from Figure S1, plasmids containing GFP (SNAP-25, syntaxin 13) and YFP (syntaxin 1) at the C-terminus were used. In the rest of this work, pN1 vectors that contain an Ochre stop codon (TAA) immediately after the Amber codon, and no FP (fluorescent protein) tag, were employed.

The plasmids encoding for SNAP-25B (pEYFP-C1) and syntaxin 1A (pEYFP-N1) were a gift from Professor Thorsten Lang (LIMES Institute, University of Bonn, Germany). SNAP-25 was subcloned into a pEGFP-N1 (Clontech) vector. Both SNAP-25 (in pEGFP-N1) and syntaxin 1 (in pEYFP-N1) constructs were subjected to site-directed mutagenesis. Their respective variants lacking the fluorescent protein moieties (pN1) were obtained by enzymatic restriction with appropriate enzymes, followed by the introduction of Ochre stop codons after the coding sequence.

The plasmid for syntaxin 13 (pET28a) was a gift from Professor Reinhard Jahn (Max Planck Institute for Biophysical Chemistry, Göttingen, Germany). The syntaxin 13 gene was directly subcloned into pEGFP-N1 and pN1 vectors containing Amber stop codons in the linker region in front of the GFP sequence (pEGFP-N1) or in the residual linker region after the enzymatic excision of GFP (pN1).

Methods

Solid Phase Peptide Synthesis

Manual SPPS of peptide **3** was performed under microwave irradiation starting with a 50 µmol scale on a Fmoc-Gly-preloaded Sieber amide resin (0.76 mmol/g). The resin was swollen in a BD syringe (Becton Dickinson, Fraga, Spain) for 2 h in DMF, and by washing with DMF (3×), DCM (3×) and NMP (3×), before starting the coupling cycle by microwave supported Fmoc-deprotection, applying 20% piperidine + 0.1 M 1-hydroxybenzotriazole (HOBt) in NMP (2×, 30 sec, 50 W, 50 °C and 3 min, 50 °C, 50 W), followed by successive washing with DMF (3×), DCM (3×) and NMP (3×). Pre-activation and coupling: The respective amino acid (5.0 eq), *O*-(benzotriazole-1-yl)-*N*,*N*,*N´*,*N´*-tetramethyluronium hexafluorophosphate (HBTU) (4.5 eq), HOBt (5.0 eq) was dissolved in NMP (0.8 mL/0.4 mL). Directly before transferring the coupling cocktail to the resin, DIEA (10 eq) was added to the pre-activated mixture. Double coupling of each amino acid (3.0 eq) with HBTU (2.7 eq), HOBt (3.0 eq) and DIEA (10 eq) was performed. 2,3,4,5,6-Pentafluorobenzoic acid was introduced under standard coupling conditions. However, to obtain better coupling yields, *O*-(7-azabenzotriazol-1-yl)-*N*,*N*,*N´*,*N´*-tetramethyluronium hexafluorophosphate (HATU) and 1-hydroxy-7-azabenzotriazole (HOAt) were used as activation reagents. Each coupling was carried out by microwave irradiation (10 min, 50 °C, 25 W) followed by washing with DMF (3×), DCM (3×), NMP (3×). After final coupling cycle, the resin was washed with DMF, DCM, NMP, DCM, MeOH, DCM (5× each) and was dried in vacuum. Cleavage of the peptide from the Sieber amide resin and simultaneous deprotection of protecting groups was obtained by using a 10% TFA/H_2O/EDT/TIS cleavage cocktail (94:2.5:2.5:1, *v*/*v*/*v*/*v*) in DCM for 1 h at ambient temperature. The reaction mixture was concentrated under nitrogen stream, followed by precipitation using cold Et_2O. The resulting suspension was centrifuged at -5 °C. The supernatant was discarded and the compound was washed with Et_2O (3×) and dried *in vacuo*. The crude peptide was purified by HPLC. The HPLC runs were performed using a linear gradient of 0.1% aq. TFA (solvent A) and 80% aq. CH_3CN/0.1% TFA (solvent B).

(F_5Bz)-K(F_5Bz)EDK(N_3)GEDKG-NH_2 (**3**) was obtained as a white solid after HPLC purification and lyophilisation. HPLC (analytical, gradient 40 → 90% B in 30 min): t_R = 12.95 min. *m/z* (ESI) = 1418.5 $[M + H]^+$, 1440.5 $[M + Na]^+$, 720.8 $[M + Na + H]^{2+}$, 1416.4 $[M - H]^-$, 707.7 $[M - 2H]^{2-}$. *m/z* (HR-ESI-MS) = calculated: 1418.4494 $[M + H]^+$, 1440.4314 $[M + Na]^+$, 1416.4349 $[M - H]^-$, found: 1418.4509 $[M + H]^+$, 1440.4318 $[M + Na]^+$, 1416.4343 $[M - H]^-$.

Synthesis of Star635-labeled peptide

(F_5Bz)-K(F_5Bz)EDK(N_3)GEDK(Star635)G-NH_2 (**SK155**)

Peptide **3** (0.55 mg, 0.39 µmol, 1.5 eq) was transferred in an Eppendorf tube, and under argon atmosphere a solution of DIEA (10 µL, 22.3 µmol, 86 eq) in dry DMF (300 µL) was added, followed by the addition of Abberior® Star635-NHS-ester (0.25 mg, 0.26 µmol, 1.0 eq) in dry DMF (25 µL), under light exclusion. After stirring for 17 h at ambient temperature, the solvent was removed *in vacuo* and the crude product was purified by HPLC. The product **SK155** (0.13 mg, 0.06 µmol, 22%) was obtained as a blue solid after lyophilisation. HPLC (analytical, gradient 40 → 90% B in 30 min): t_R = 21.74 min. *m/z* (ESI) = 1181.9 $[M + 3Na]^{2+}$, 1146.8 $[M - H]^{2-}$. *m/z* (HR-ESI-MS) = calculated: 1181.8367 $[M + 3Na]^{2+}$, 787.8926 $[M + 3Na]^{3+}$, found: 1181.8350 $[M + 3Na]^{2+}$, 787.8913 $[M + 3Na]^{3+}$.

Site-directed mutagenesis. Site-directed mutagenesis primers were designed to introduce Amber stop codons (TAG) using the QuikChange Site-Directed Mutagenesis Kit II (Stratagene); protocols were applied according to the instructions of the manufacturer. Consult Figure S1 for more details on the positions of the mutations.

^{15}N-L-Leucine and propargyl-L-lysine (PRK) incorporation. BHK cells were grown for 2 days in 200 mg/mL ^{15}N-L-leucine (20 mg/mL stock in PBS) in leucine- and antibiotic-free DMEM supplemented with 5% dialyzed serum and 2 mM L-glutamine (^{15}N-L-leucine medium). On the third day, the medium was exchanged to ^{15}N-L-leucine medium containing 250 µM propargyl-L-lysine (PRK, dissolved as a 1 M stock in DMSO) prior to transfection. PRK incorporation was carried out by co-transfecting cells with two vectors: pCMV tRNA-Pyl RS WT (encoding for the bioorthogonal aminoacyl-tRNA-synthetase/tRNA pair)[5] and the vectors encoding for the proteins of interest (SNAP-25, syntaxin 1, and syntaxin 13). Transfection using Lipofectamine 2000 Reagent (Life Technologies) was performed by first equilibrating for 5 min plasmids and Lipofectamine with Opti-MEM (Gibco) in separate Eppendorf tubes, and subsequently mixing the two and incubating them for 20 min. The mixture was applied on the cells, which were incubated at 37 °C for 18 h. Refer to Figure S5 for an overview of the experimental timeline. The medium was exchanged to normal DMEM 2 h prior to fixation.

Click reaction. Unless otherwise stated, samples were fixed with 0.2% glutaraldehyde and 4% PFA in PBS (phosphate-buffered saline: 137 mM NaCl, 2.7 mM KCl, 10 mM Na_2HPO_4, 2 mM KH_2PO_4, pH 7.3). Next, a 20-minute quenching step in 100 mM NH_4Cl and 100 mM glycine (in PBS) was performed, followed by a brief wash with PBS. Samples were then permeabilized with PBS containing 0.1% Triton X-100, and then blocked for 15 minutes with PBS containing 0.1% Triton-X 100, 5% BSA and 5% peptone. The cells were briefly incubated with PBS containing 3% BSA prior to the click reaction. The click incubation mixture was prepared shortly before adding it to the cells, following the manufacturer's protocol (Click-iT® Cell Reaction Buffer Kit, Life Technologies, cat. no. C10269). It contained 1x cell reaction buffer (component A), 2 mM $CuSO_4$ (component B), a 10× dilution of Click-iT reaction buffer additive (component C), and 7 µM final concentration of **SK155** (in DMSO) or 50 µM Star635P-azide (in PBS). The cells were incubated with the reaction mix for 30 minutes at RT, in a dark humidified chamber.

DAPI labeling of nuclei. Optionally, the cells were washed 3 times with 5% BSA and 5% peptone in PBS and 3 times with PBS after the click reaction. Then, cell nuclei were stained with 1 µg/mL DAPI for 5 minutes, followed by three additional PBS washes, before embedding in Mowiol mounting medium (24% w/v glycerol, 0.1M Tris-HCl, pH 8.5, 9.6% w/v Mowiol 4-88; Carl Roth GmbH, Karlsruhe, Germany).

Immunostaining. After the click reaction, cells were washed with 0.1% Triton X-100 in PBS containing 1.5% BSA (3 solution exchanges, 5 minutes each), and then subjected to immunostaining for 1 hour with anti-calnexin (AbCam, Cambridge, UK) primary antibody. The 1.5% BSA/PBS washing steps were repeated before the 1-hour incubation with Cy2-labeled anti-rabbit antibody. Both primary and secondary antibodies were used at a dilution of 1:100 in PBS with 1.5% BSA and 0.1% Triton X-100. After immunostaining, the cells were subjected to two PBS washes, one high-salt PBS wash, followed by another PBS wash (5 minutes each).

Plastic embedding of whole cells, and thin sectioning. The cells were post-fixed with 0.2% glutaraldehyde-4% PFA in PBS, for 30 min. Two short PBS washes were performed before a 20-minutes quenching step, with 100 mM NH_4Cl and 100 mM glycine in PBS. Three more PBS washing steps (5 minutes each) were carried out before proceeding to embedding.

The samples were embedded in LR White resin (London Resin Company Ltd, Berkshire, England) as previously described[6]. The LR White-embedded samples where cut using an EM UC6 ultramicrotome (Leica Microsystems) into 200 nm samples that were then mounted on silicone wafers.

Confocal microscopy. Multichannel confocal images were taken with a Leica TCS SP5 microscope (Leica Microsystems) using a 100× 1.4 N.A. HCX PL APO CS oil objective (Leica). To visualize the fluorophores, the following excitation lines were employed: an argon laser at a wavelength of 488 nm for Cy2, and a helium-neon laser at 633 nm for Star635. An AOTF filter (Leica) was used to select appropriate emission intervals. Signal detection was performed using photomultiplier tubes. Images were acquired at 7.5× zoom by scanning sequentially line-by-line at 1,000 Hz the two channels. Final images have a 1024×1024 pixels format with pixel size of 20.21×20.21 nm (for a 20.68×20.68 µm scanned area).

Secondary Ion Mass Spectrometry (SIMS). NanoSIMS measurements were performed using a NanoSIMS 50L instrument (Cameca, Gennevilliers, France), installed at TUM Freising. Samples were sputtered using a fine focused Cs^+ primary ion beam at 16 keV energy. The resulting secondary ions were separated by mass and detected by electron multipliers. Measurements were performed with a primary current of ~1-2 pA, at an estimated lateral resolution of ~100 nm. The mass resolving power was set to reach a reliable separation of the potential isobars. Before the measurement, a pre-sputtering with a high primary current was performed for the implantation of Cs^+ ions promoting the ionization efficiency. Sample areas of 20×20 µm were scanned with 512×512 pixel and a 10 ms dwell-time per pixel. Unless otherwise stated, figures represent single plane measurements. When multiple planes were measured, image accumulation was done using a Matlab routine. To image the distribution of relevant species, the following ions were detected: $^{19}F^-$, $^{12}C^{14}N^-$, $^{12}C^{15}N^-$, referred through the manuscript as ^{19}F, ^{14}N and ^{15}N, respectively. For presentation purposes, the images were binned using a 2x2 binning procedure.

Epifluorescence Microscopy. An Olympus IX 71 inverted fluorescence microscope (Olympus, Hamburg, Germany) equipped with a 20× objective (Olympus, 0.50 N.A.) was employed for fluorescence visualization. Samples were illuminated by a 100 W mercury lamp (Olympus) and images of fluorescently labeled cells were captured by a computer-operated (CellF software, Olympus) charge-coupled device (CCD) camera (FView II, Olympus).

Fluorescence in the DAPI channel was detected using a 377/50 excitation filter, 409 long pass beam splitter and 447/60 emission filter. For the green channel (GFP or YFP/FITC), the following filters were used: 480/40 HQ excitation filter, 505 LP Q beam splitter, and 527/30 HQ emission filter. In the red channel, Abberior Star635P fluorescence was detected using the 620/60 HQ excitation filter, the 660 LP Q beam splitter, and the 700/75 HQ emission filter. All filters were produced by AHF (Tübingen, Germany).

Data analysis. All analyses were performed using self-written routines in Matlab (the Mathworks Inc, Natick, MA). The fluorescence and SIMS images were overlaid and corrected for drifts and for rotational misalignments. Circular regions of 9 pixels in diameter were then collected manually in the different images. The average fluorescence intensity, or the average isotopic counts, were determined, and plotted in Figures 3 and 4. In Figure 3B the ratio between ^{19}F and ^{14}N was used, rather than the raw levels of ^{19}F, since it is less subjected to experimental variations in NanoSIMS imaging. We would also like to point out that the analysis of ratios, rather than absolute levels, is a routine procedure in SIMS.

Statistics. The student's t-test was used to assess the difference between the $^{19}F/^{14}N$ ratios in transfected versus non-transfected cells (Figure 3B).

Supporting Figures

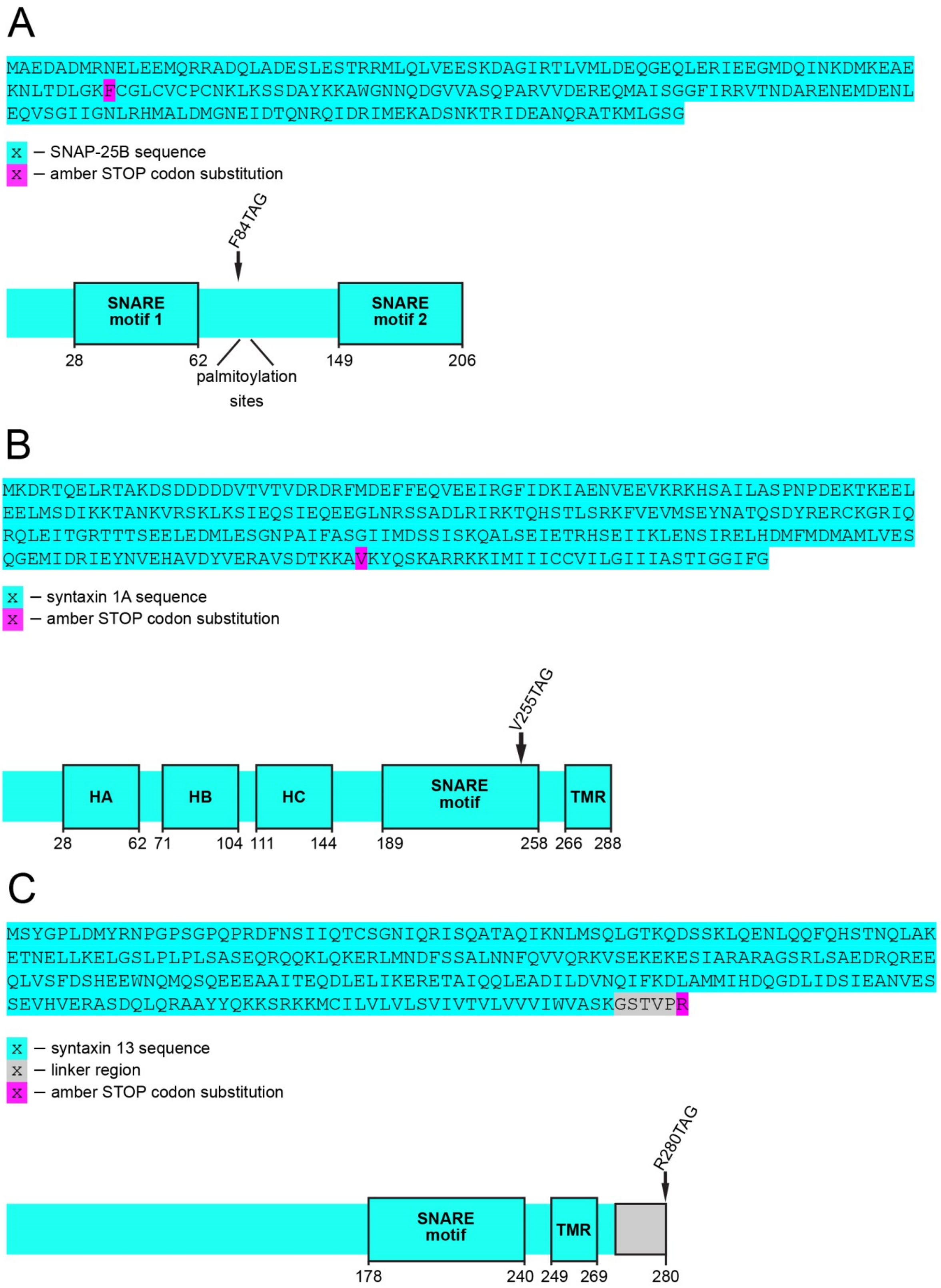

Figure S1: DNA constructs. Amino acid sequences of wild-type SNAP-25B (Uniprot primary accession number: P60881; RefSeq NP_112253.1; **A),** syntaxin 1A (P32851; NP_446240.2; **B**), and syntaxin 13 (G3V7P1; NP_075228.2; **C**), and their corresponding

schematic representations. The sequences of the proteins of interest are depicted in light blue. Linker regions are shown in light grey. Magenta shading is used to highlight positions where codons were replaced with Amber stop codons, via site-directed mutagenesis. The SNAP-25 and syntaxin 1 constructs were generated by enzymatic restriction from vectors encoding for FP at the C-terminus of each protein, followed by re-ligation and site-directed mutagenesis of the products, to encode for an Ochre stop codon (TAA) after the full-length coding sequence of the protein. Syntaxin 13 was directly cloned into vectors pEGFP-N1 and pN1 in such a manner that the final constructs presented an Amber stop codon and a GFP at the C-terminus (pEGFP-N1) or an Ochre codon immediately followed the Amber stop codon at position 280 (pN1).

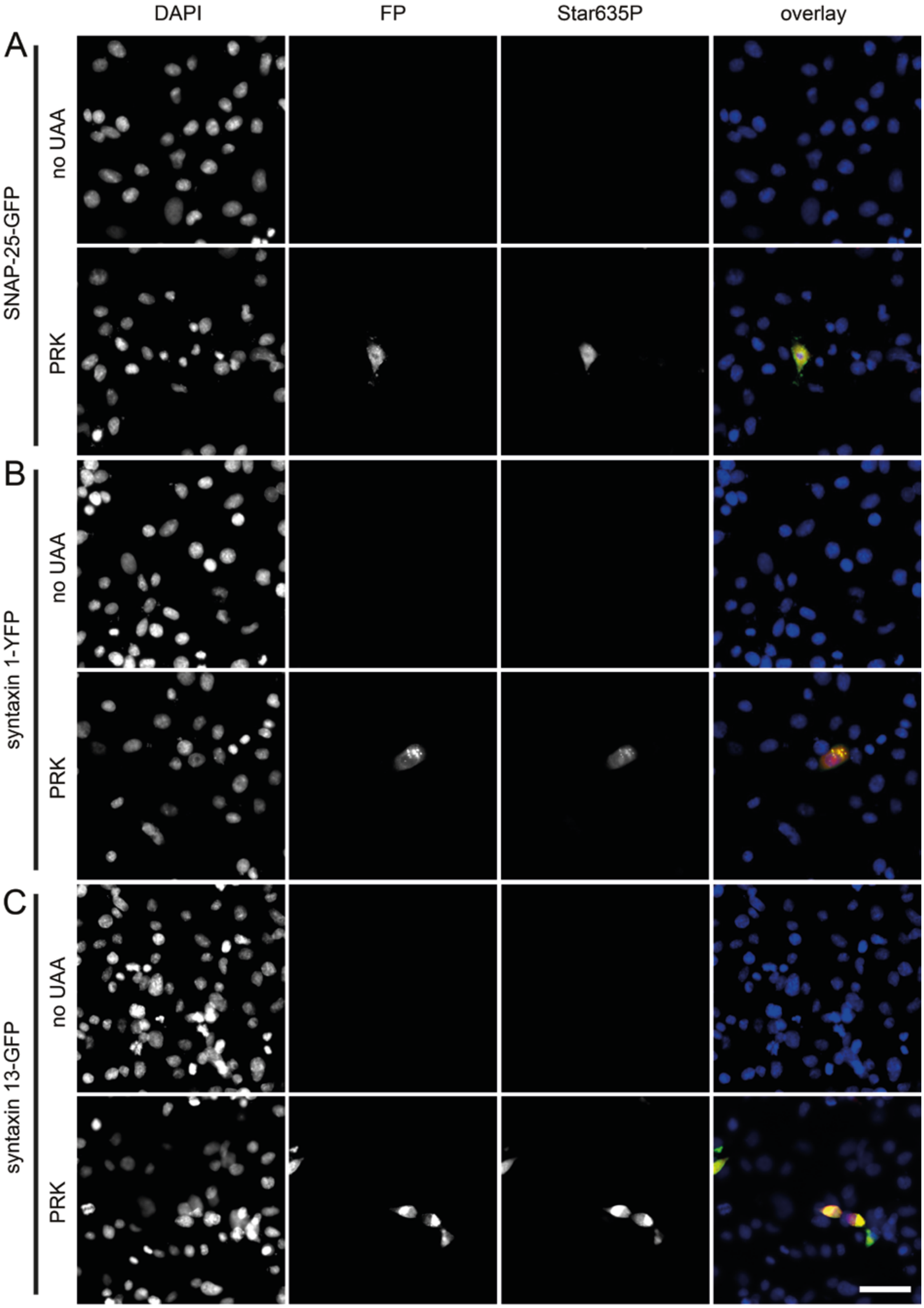
DAPI
FP
Star635P
overlay
A
SNAP-25-GFP
no UAA
PRK
B
syntaxin 1-YFP
no UAA
PRK
C
syntaxin 13-GFP
no UAA
PRK

Figure S2: Click reaction is specific. High specificity of click reactions for SNAP-25, syntaxin 1, and syntaxin 13. Typical images of BHK cells co-transfected with the tRNA/RS pair from *Methanosarcina mazei* (pCMV vector), and with vectors encoding for constructs containing the Amber codon, encoding for SNAP-25-GFP (A), syntaxin 1-YFP (B), and syntaxin 13-GFP (C). The cells were allowed to express the proteins of interest and to incorporate the unnatural amino acid PRK. Controls (no UAA) were similarly transfected but were deprived of PRK. The cells were afterwards labeled with the red fluorophore Star635P by copper-catalyzed click chemistry. The click reaction, detected by Star635P fluorescence, is specific for the cells expressing Amber- and FP-containing proteins. In the overlay images DAPI-stained nuclei are shown in blue, fluorescent protein (FP) signal is green, while Star635P signal is red. Scale bar, 40 µm.

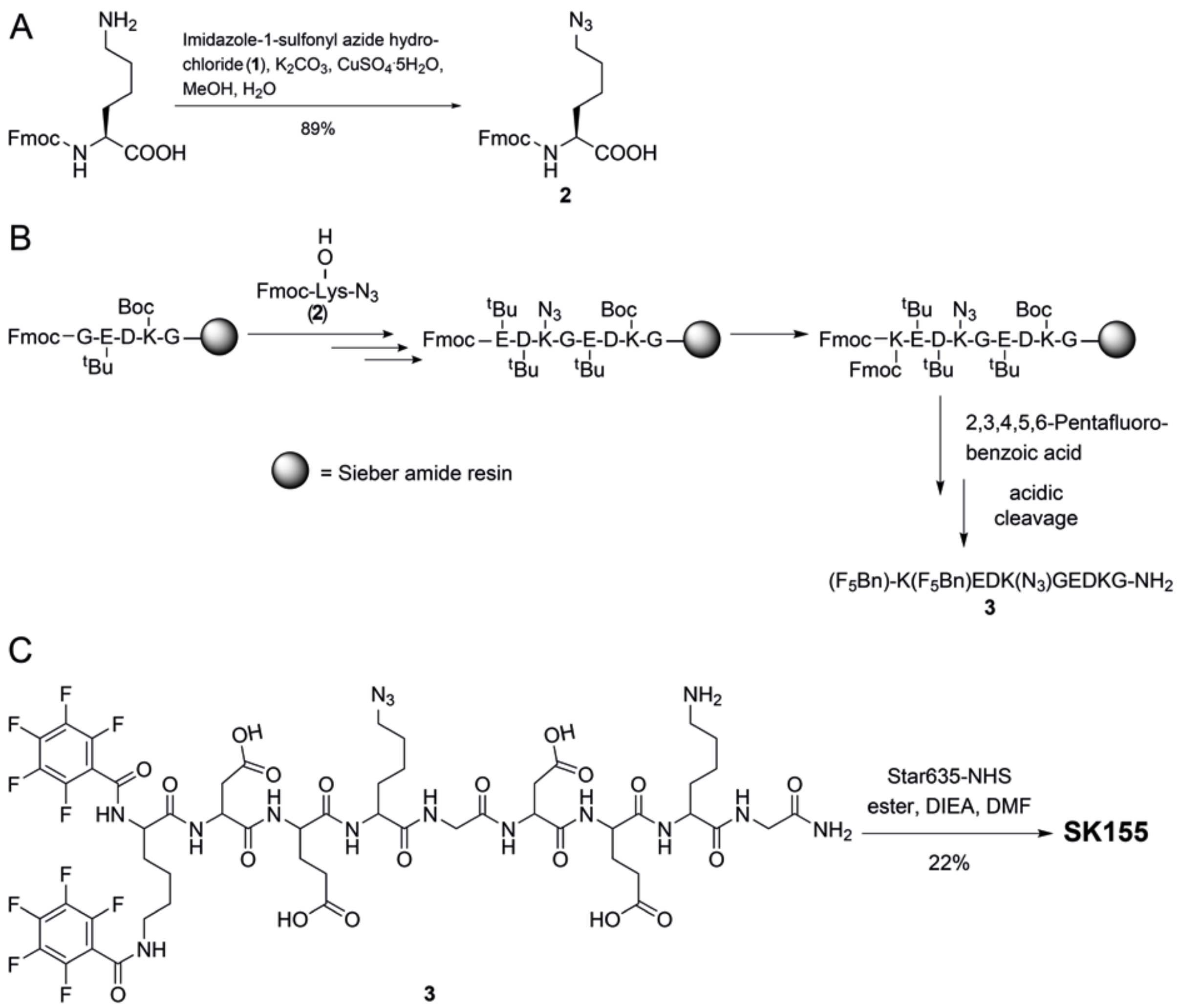

Figure S3: SK155 synthesis. (**A**) Starting from commercially available Fmoc-Lys-OH, Fmoc-Lys(N_3)-OH[2,3] (**2**) was synthesized by a copper(II) catalayzed diazo transfer reaction with previously prepared 1*H*-imidazole-1-sulfonyl azide hydrochloride[1,2] (**1**) as an azide transfer reagent. (**B**) Fmoc-Lys(N_3)-OH (**2**) was incorporated by manual solid phase peptide synthesis (SPPS) under microwave irradiation, following the Fmoc-protocol on Sieber amide resin. 2,3,4,5,6-Pentafluorobenzoic acid was introduced following standard coupling conditions.[7] Cleavage from the resin and simultaneous removal of protecting groups yielded crude peptide **3**, which was purified by HPLC and characterized by high-resolution mass spectrometry. (**C**) Labeling of peptide **3** with Star635 NHS ester was accomplished under light exclusion in solution to yield **SK155**. This compound was purified by HPLC and characterized by high-resolution mass spectrometry.

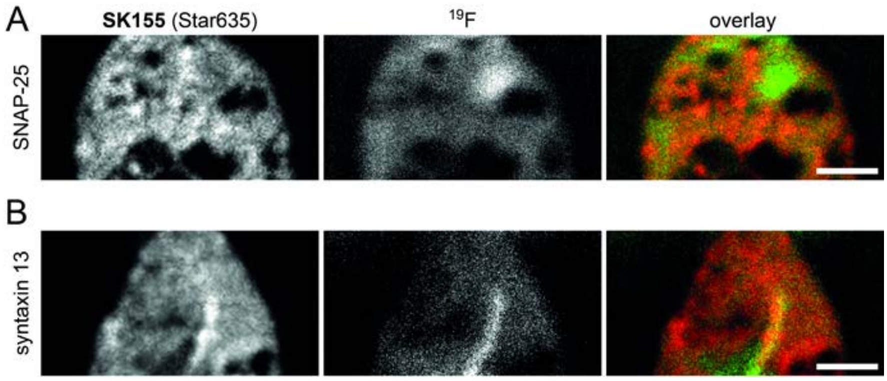

Figure S4: Genetic encoding of PRK into SNAP-25 and syntaxin 13. Fluorescence (**SK155**), NanoSIMS (^{19}F), and overlay (**SK155** shown in red, and ^{19}F in green) images for representative cells expressing SNAP-25 (**A**) and syntaxin 13 (**B**). Scale bar, 2 µm.

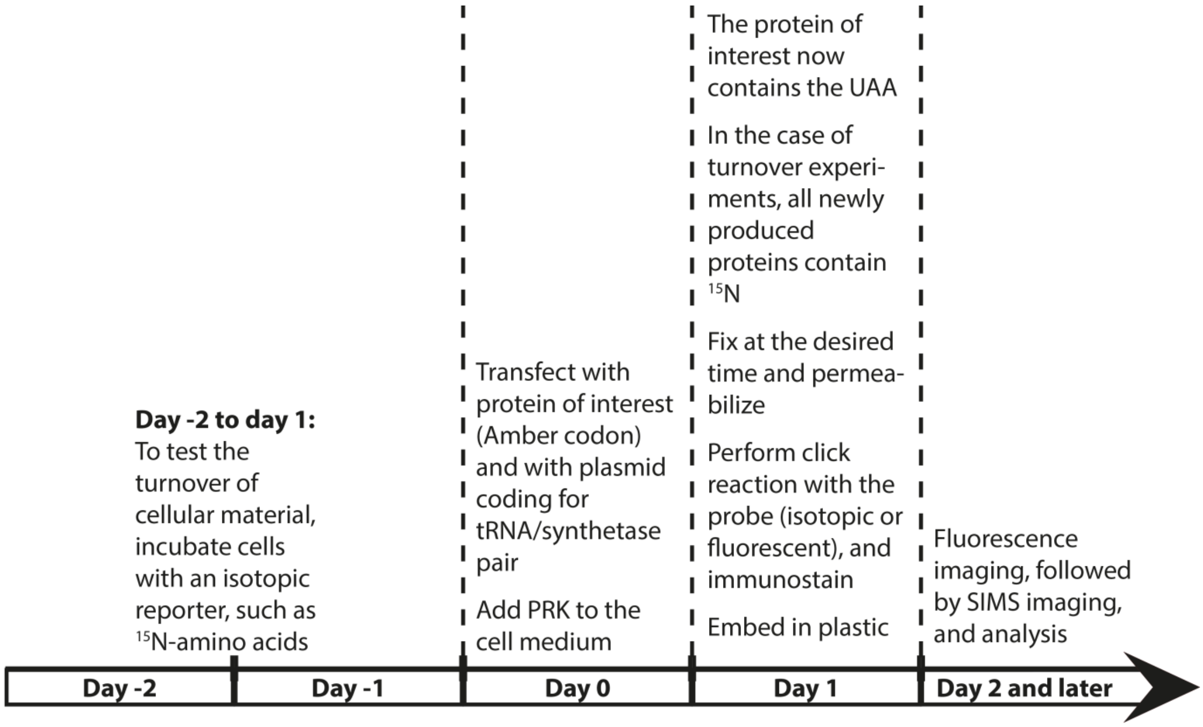

Figure S5: Experimental timeline.

References

[1] C. Byrne, P. a McEwan, J. Emsley, P. M. Fischer, W. C. Chan, *Chem. Commun. (Camb).* **2011**, *47*, 2589–2591.

[2] E. Sabidó, T. Tarragó, E. Giralt, *Bioorg. Med. Chem. Lett.* **2009**, *19*, 3752–3755.

[3] E. D. Goddard-Borger, R. V Stick, *Org. Lett.* **2007**, *9*, 3797–3800.

[4] K. Kolmakov, V. N. Belov, J. Bierwagen, C. Ringemann, V. Müller, C. Eggeling, S. W. Hell, *Chemistry* **2010**, *16*, 158–166.

[5] T. Plass, S. Milles, C. Koehler, J. Szymański, R. Mueller, M. Wiessler, C. Schultz, E. A. Lemke, *Angew. Chem. Int. Ed. Engl.* **2012**, *51*, 4166–70.

[6] S. K. Saka, A. Vogts, K. Kröhnert, F. Hillion, S. O. Rizzoli, J. T. Wessels, *Nat. Commun.* **2014**, *5*, 3664.

[7] K. Tomita, S. Oishi, H. Ohno, N. Fujii, *Biopolymers* **2008**, *90*, 503–511.
